# Identification of crucial noncoding RNAs and mRNAs in hypertrophic scars via RNA sequencing

**DOI:** 10.1002/2211-5463.13167

**Published:** 2021-05-12

**Authors:** Xiaodong Li, Zeliang He, Julei Zhang, Yan Han

**Affiliations:** ^1^ Department of Burn and Plastic Surgery The 980st Hospital of the PLA Joint Logistics Support Force Shijiazhuang China; ^2^ Department of Plastic and Reconstructive Surgery The First Medical Center Chinese PLA General Hospital Beijing China

**Keywords:** ceRNA network, circRNA, hypertrophic scar, lncRNA

## Abstract

Hypertrophic scarring (HS) is a dermal fibroproliferative disorder characterized by excessive deposition of collagen and other extracellular matrix components. The aim of this study is to explore crucial long noncoding RNAs (lncRNAs) and circular RNAs (circRNAs) associated with HS and provide a better understanding of the molecular mechanism of HS. To investigate the lncRNA, circRNA and mRNA expression profiles, we performed RNA sequencing of human HS and normal skin tissues. After the identification of differentially expressed mRNAs (DEmRNAs), lncRNAs (DElncRNAs) and circRNAs (DEcircRNAs), we performed functional enrichment of DEmRNAs. Further on, we constructed DElncRNA/DEcircRNA–DEmRNA coexpression networks and competing endogenous RNA regulatory networks, and performed functional analyses of the DEmRNAs in the constructed networks. In total, 487 DEmRNAs, 92 DElncRNAs and 17 DEcircRNAs were identified. DEmRNAs were significantly enriched in processes such as collagen fibril organization, extracellular matrix–receptor interaction and the phosphatidylinositol 3‐kinase (PI3K)–Akt signaling pathway. In addition, we detected 580 DElncRNA–DEmRNA and 505 DEcircRNA–DEmRNA coexpression pairs. The competing endogenous RNA network contained 18 circRNA–microRNA (miRNA) pairs, 18 lncRNA–miRNA pairs and 409 miRNA–mRNA pairs, including 10 circRNAs, 5 lncRNAs, 15 miRNAs and 160 mRNAs. We concluded that MIR503HG/hsa‐miR‐204‐3p/*ACAN*, MIR503HG/hsa‐miR‐431‐5p/*TNFRSF9*, MEG3/hsa‐miR‐6884‐5p/*ADAMTS14*, AC000035.1‐*ADAMTS14* and hsa_circ_0069865‐*COMP*/*ADAM12* interaction pairs may play a central role in HS.

## Graphical abstract

We performed RNA sequencing to acquire the expression profiles of long noncoding RNA, circular RNA and mRNA in human hypertrophic scar (HS) and normal skin tissues from three male patients with HS. We concluded that MIR503HG/hsa‐miR‐204‐3p/*ACAN*, MIR503HG/hsa‐miR‐431‐5p/*TNFRSF9*, MEG3/hsa‐miR‐6884‐5p/*ADAMTS14*, AC000035.1‐*ADAMTS14* and hsa_circ_0069865‐*COMP*/*ADAM12* interaction pairs may play a central role in HS.

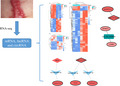

**Abbreviations**
ADAM: a disintegrin and metalloprotease
CD1A: CD1a molecule
ceRNA: competing endogenous RNA
circRNA: circular RNA
DEcircRNA: differentially expressed circRNA
DElncRNA: differentially expressed lncRNA
DEmRNA: differentially expressed mRNA
ECM: extracellular matrix
FC: fold change
GO: Gene Ontology
HCC: hepatocellular carcinoma
HS: hypertrophic scar
KEGG: Kyoto Encyclopedia of Genes and Genomes
lncRNA: long noncoding RNA
miRNA: microRNA
OSCC: oral squamous cell carcinoma

Scar formation is an inevitable result of wound healing. Hypertrophic scarring (HS), a type of pathological scarring, with a protruding surface, irregular shape and burning and itching sensations on the skin surface, often significantly affects patients' quality of life [1]. HS is characterized by excessive deposition and alterations in morphology of collagen and other extracellular matrix (ECM) proteins [2]. Clinically, it is identified by excessive dermal fibrosis and scarring resulting from the imbalance between collagen synthesis and degradation during wound healing [3]. Although numerous interventions for HS, including surgical removal, radiotherapy, steroid injection and cryotherapy, are available, these treatments cannot achieve a stable curative effect [4]. The etiology and pathogenesis of HS have been explored for decades, but the molecular mechanisms of HS remain poorly understood [5]. Therefore, it is of great importance to elucidate the mechanisms of HS and explore new targets for treatment of HS.

Long noncoding RNAs (lncRNAs) and microRNAs (miRNAs), as novel noncoding RNAs, have been reported to be involved in HS. For instance, Li *et al*. [6] indicated that up‐regulated lncRNA8975‐1 in HS fibroblasts inhibited fibroblast proliferation and reduced collagen expression. Nong *et al*. [7] demonstrated that lncRNA COL1A2‐AS1 inhibited fibroblasts proliferation to suppress HS formation via regulating the miR‐21–Smad7 pathway. Wu *et al*. [8] reported that miR‐155 inhibited the formation of HS fibroblasts by targeting hypoxia induciblefactor 1 subunit alpha (HIF‐1α) via the PI3K–AKT pathway. Shen *et al*. [9] suggested that miR‐145‐5p arrested the development of fibrogenesis and decreased HS formation by reducing the expression of Smad2/3. Zhang *et al*. [10] found that miR‐137 inhibited proliferation and metastasis of HS fibroblasts via targeting pleiotrophin. However, to the best of our knowledge, there was only one study exploring the expression profiles of circular RNA (circRNA) in HS [11].

This study investigated the mRNA, lncRNA and circRNA expression profiles of HS to identify the differentially expressed mRNAs (DEmRNAs), lncRNAs (DElncRNAs) and circRNAs (DEcircRNAs) associated with HS. In addition, a competing endogenous RNA (ceRNA) (DEcircRNA/lncRNA–miRNA–DEmRNA) regulatory network was conducted. This study sought to make a contribution to elucidating the potential molecular mechanisms of HS and lay a foundation for the treatment of HS.

## Materials and methods

### Subjects and samples

HSs and adjacent normal skin tissues were collected from three male patients in our hospital, aged 6, 21 and 22 years. The site of HS was neck, left arm and right arm. All samples were collected after obtaining written informed consent from every participant. This study was approved by the ethics committee of The 980st Hospital of the PLA Joint Logistics Support Force (2020‐KY‐25) and performed in accordance with the Declaration of Helsinki. Total RNAs were isolated from HS and normal skin tissues with TRIzol reagent. Based on the Illumina HiSeq X‐ten platform, sequencing was performed.

### Quality control of raw sequencing and mapping of clean reads

To obtain clean reads from RNA sequencing results, we removed sequences with low quality, including adapter sequences, sequences with quality score < 20, sequences with N base rate of raw reads > 10% and sequence < 25 bp. Hisat2 was used to align clean reads with the human reference genome Ensemble GRCh38. Expression of mRNAs and lncRNAs was normalized and outputted with StringTie. Then, ciri2 software was used to predict circRNAs.

### Identification of DEmRNAs, DElncRNAs and DEcircRNAs

Ballgown was applied to identify DEmRNAs, DElncRNAs and DEcircRNAs in HS with |log_2_FC| > 1 and *P* < 0.05. Hierarchical clustering analysis of DEmRNAs, DElncRNAs and DEcircRNAs was performed with r (https://www.r‐project.org/) package ‘pheatmap’. David 6.8 was used to perform Gene Ontology (GO) and Kyoto Encyclopedia of Genes and Genomes (KEGG) enrichment analysis for DEmRNAs with *P* < 0.05.

### DElncRNA/DEcircRNA–DEmRNA coexpression network

The DElncRNA/DEcircRNA–DEmRNA coexpression network was constructed to further investigate the potential functions of lncRNAs, circRNAs and mRNAs in HS. The lncRNA–mRNA pairs with |*r*| > 0.99 and *P* < 0.05 were defined as coexpressed DElncRNA–DEmRNA pairs, and circRNA–mRNA pairs with |*r*| ≥ 0.95 and *P* < 0.05 were defined as coexpressed DEcircRNA–DEmRNA pairs, respectively. Then, coexpressed networks were visualized by using cytoscape. david 6.8 was used to perform GO and KEGG enrichment analysis for DEmRNAs in the DElncRNA/DEcircRNA–DEmRNA coexpression network with *P* < 0.05.

### ceRNA (DEcircRNA/DElncRNA–miRNA–DEmRNA) regulatory network

Based on the results of the differential expression analysis, DEcircRNA–miRNA interaction pairs were predicted with the CircBank (http://www.circbank.cn/index.html) database. The targeted DEmRNAs of miRNAs were predicted with miRWalk 3.0 (http://mirwalk.umm.uni‐heidelberg.de/). The DElncRNA–miRNA interaction pairs were predicted with NPInter v4.0 (http://bigdata.ibp.ac.cn/npinter4/). Then, the ceRNA (DEcircRNA/DElncRNA–miRNA–DEmRNA) regulatory network was constructed by combining circRNA/lncRNA–miRNA pairs with miRNA–mRNA pairs. Ultimately, cytoscape was used to visualize the regulatory network. With david 6.8, GO and KEGG pathway analyses of all DEmRNAs in the ceRNA regulatory network were performed. Statistical significance was defined as *P* < 0.05.

## Results

### Identification of DEmRNAs, DElncRNAs and DEcircRNAs

**Fig. 1:**
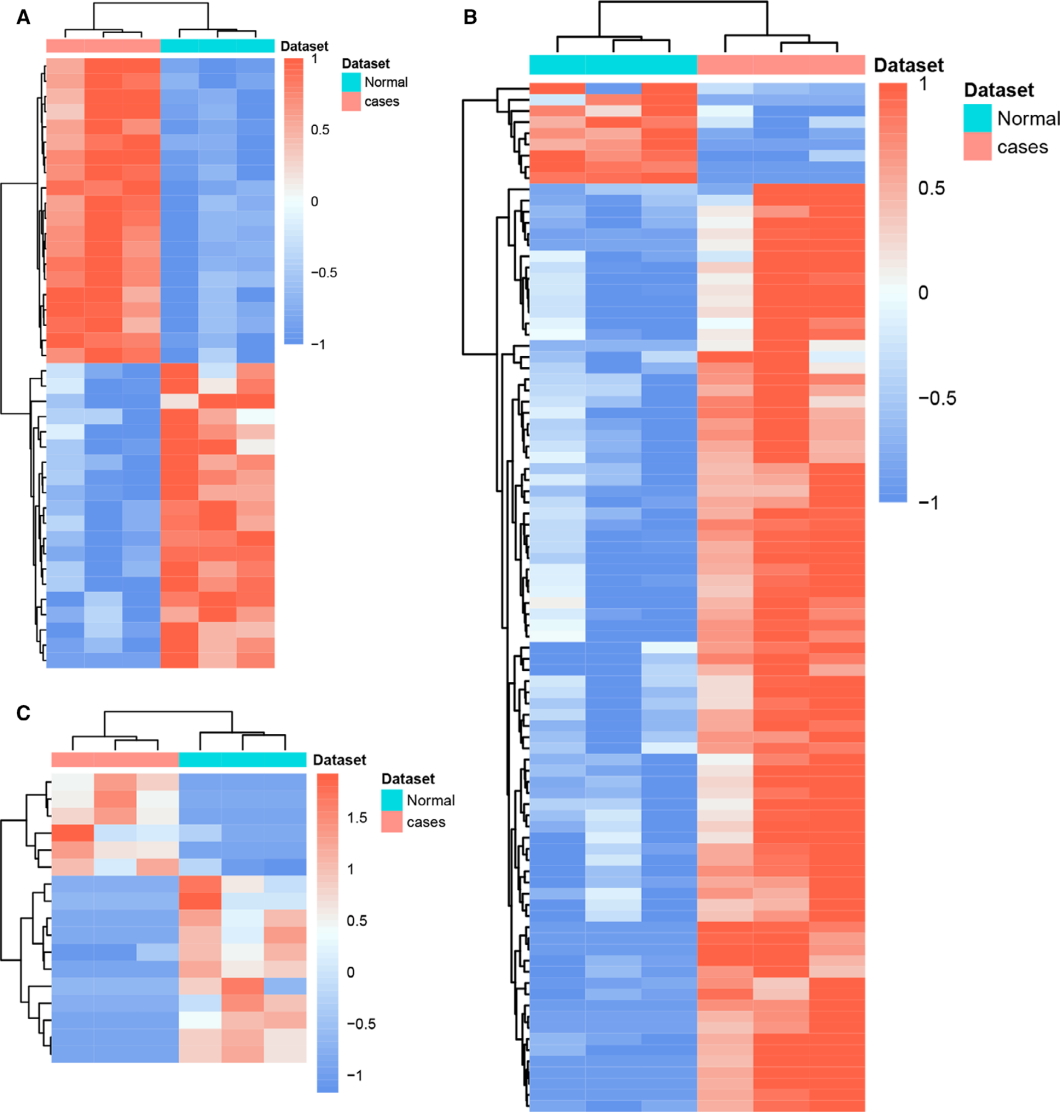
Unsupervised hierarchical clustering analysis of the DEmRNAs (A), DElncRNAs (B) and DEcircRNAs (C) between HS and adjacent normal skin tissues. Row and column represented DEmRNAs/DElncRNAs/DEcircRNAs and tissue samples, respectively. The color scale represented the expression levels. *n* = 3.

Compared with adjacent normal skin tissues, 487 DEmRNAs (457 up‐ and 30 down‐regulated), 92 DElncRNAs (83 up‐ and 9 down‐regulated) and 17 DEcircRNAs (6 up‐ and 11 down‐regulated) were identified in HS. Of these, *ACAN* and CD1a molecule (*CD1A*), AC105105.1 and LINC01189, and hsa_circ_0008667 and hsa_circ_0021727 were the most up‐ and down‐regulated DEmRNA/DElncRNA/DEcircRNA in HS, respectively (Table 1). The heatmap of the DEmRNAs/DElncRNAs/DEcircRNAs was shown in Fig. 1.

**Table 1:** Top 10 up‐ and down‐regulated DEmRNAs/DElncRNAs/DEcircRNAs in HS. FDR, false discovery rate.

Symbol	log_2_FC	*P* Value	FDR	Regulation
mRNA				
*ACAN*	7.341256	3.97E−60	6.20E−56	Up
*ADAM12*	5.949145	2.74E−31	2.15E−27	Up
*NEFH*	4.289851	5.75E−23	3.00E−19	Up
*ADAMTS16*	3.971908	1.19E−21	4.64E−18	Up
*COMP*	4.899795	5.04E−21	1.58E−17	Up
*COL11A1*	5.500081	1.44E−19	3.76E−16	Up
*TNFRSF9*	4.622602	3.53E−19	7.87E−16	Up
*ADAMTS14*	5.942227	4.87E−19	9.52E−16	Up
*P4HA3*	5.473385	1.61E−18	2.80E−15	Up
*ANOS1*	2.446834	4.34E−17	6.78E−14	Up
*CD1A*	−2.16518	2.36E−7	5.20E−5	Down
*IGFL4*	−3.29682	1.68E−6	0.000258	Down
*SPRR4*	−5.84497	2.31E−6	0.000329	Down
*EDA*	−1.75118	4.91E−6	0.000635	Down
*AKAP1*	−1.91416	5.39E−6	0.000678	Down
*CD207*	−2.25162	9.51E−6	0.001039	Down
*ECHDC3*	−2.19145	1.60E−5	0.001452	Down
*ARL2‐SNX15*	−6.03628	1.60E−5	0.001452	Down
*RORA*	−1.13228	2.16E−5	0.001855	Down
*VSIG8*	−3.71747	5.54E−5	0.003919	Down
lncRNA				
AC105105.1	4.524189	7.20E−16	6.14E−12	Up
MIR503HG	4.492636	1.03E−13	4.40E−10	Up
LINC01614	7.15706	1.39E−11	3.97E−8	Up
LINC01561	4.411686	5.80E−9	1.07E−5	Up
LINC01429	5.853049	6.28E−9	1.07E−5	Up
LINC01711	4.803794	1.28E−8	1.83E−5	Up
LINC02544	5.890596	1.91E−8	2.07E−5	Up
AC090197.1	2.184541	1.94E−8	2.07E−5	Up
AC113383.1	3.404982	8.88E−8	8.31E−5	Up
AC000035.1	12.3098	1.01E−7	8.31E−5	Up
LINC01189	−8.11434	1.31E−6	0.000558	Down
AL031291.1	−11.3124	6.09E−6	0.001584	Down
AC010329.1	−3.44806	5.46E−5	0.008625	Down
LINC01290	−2.22062	9.21E−5	0.012892	Down
BX088651.2	−4.76479	0.000276	0.029441	Down
LINC00842	−2.43539	0.000286	0.030118	Down
LINC02242	−9.60651	0.000316	0.032302	Down
LINC00205	−3.39688	0.000382	0.037073	Down
AC245123.1	−7.85624	0.000434	0.041644	Down
circRNA				
hsa_circ_0008667	7.399127	0.033936	0.855118	Up
hsa_circ_0000699	7.325837	0.03786	0.855118	Up
hsa_circ_0006370	3.963574	0.037886	0.855118	Up
hsa_circ_0004751	7.244633	0.039784	0.855118	Up
hsa_circ_0002874	2.802046	0.045302	0.855118	Up
hsa_circ_0007755	7.067075	0.046354	0.855118	Up
hsa_circ_0021727	−8.12769	0.005618	0.855118	Down
hsa_circ_0000826	−8.41942	0.005818	0.855118	Down
hsa_circ_0069865	−7.72155	0.010411	0.855118	Down
hsa_circ_0085173	−7.46746	0.019994	0.855118	Down
hsa_circ_0000650	−7.36772	0.022593	0.855118	Down
hsa_circ_0008172	−7.12402	0.035404	0.855118	Down
hsa_circ_0007108	−7.41823	0.037163	0.855118	Down
hsa_circ_0006956	−7.03894	0.042358	0.855118	Down
hsa_circ_0005239	−3.63413	0.04609	0.855118	Down
hsa_circ_0006987	−6.83046	0.049686	0.855118	Down

### Functional annotation of DEmRNAs

**Fig. 2:**
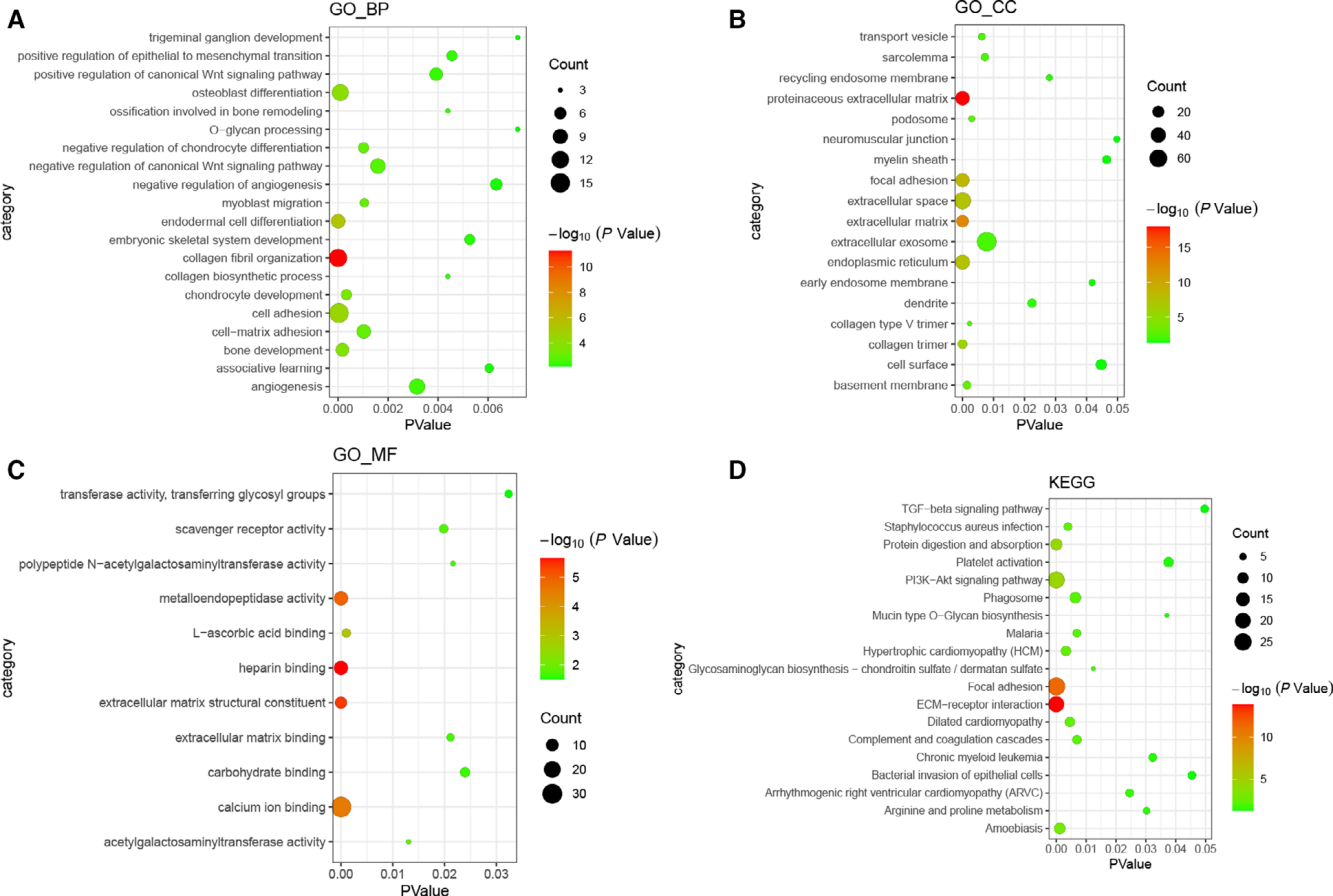
Significantly enriched GO terms and KEGG pathways of DEmRNAs in HS. (A) BP, biological process; (B) CC, cellular component; (C) MF, molecular function; (D) KEGG pathways. The *x* axis shows *P* value of GO terms or KEGG pathways, and the *y* axis shows GO terms or KEGG pathways. The color scale represented −log *P* value.

GO enrichment analysis revealed that biological processes such as collagen fibril organization (*P* = 5.48E−12), cell adhesion (*P* = 3.47E−5), proteinaceous ECM (*P* = 1.11E−18), heparin binding (*P* = 2.17E−6) and ECM structural constituent (*P* = 3.90E−6) were dysregulated (Fig. 2A–C). KEGG pathway analysis highlighted that ECM–receptor interaction (*P* = 1.35E−14), focal adhesion (*P* = 4.32E−12), PI3K–Akt signaling pathway (*P* = 2.53E−5) and protein digestion and absorption (*P* = 3.74E−5) were dysregulated (Fig. 2D).

### DElncRNA–DEmRNA coexpression network

**Fig. 3:**
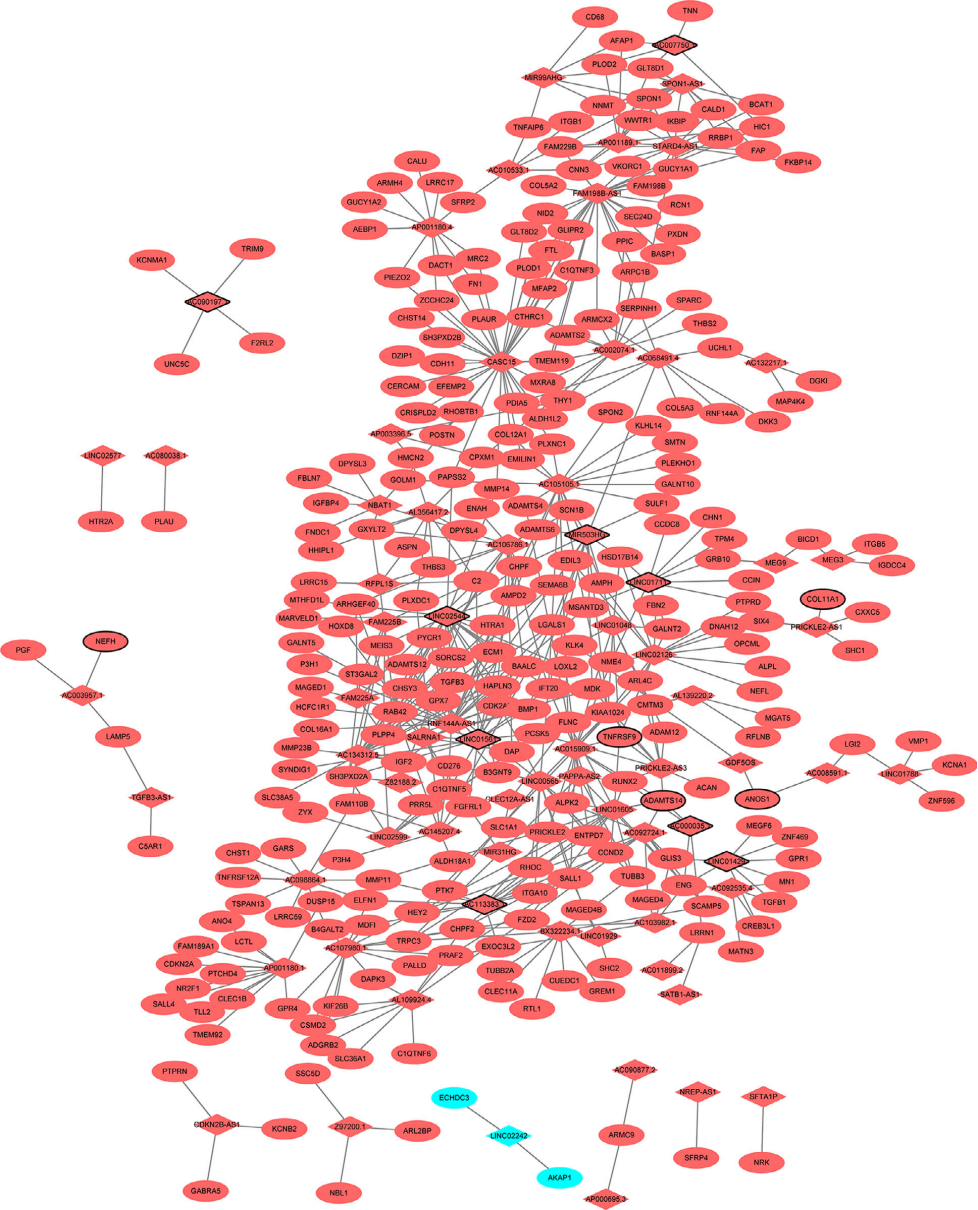
DElncRNA–DEmRNA coexpression network. The rhombuses and ellipses represent DElncRNAs and DEmRNAs, respectively. Red and blue colors represent up‐ and down‐regulation, respectively. Nodes with black border were DElncRNAs/DEmRNAs derived from top 10 up‐ and down‐regulated DElncRNAs/DEmRNAs in HS.

A total of 580 DElncRNA–DEmRNA coexpression pairs, including 72 DElncRNAs and 277 DEmRNAs, were obtained (Fig. 3). Among these lncRNAs, CASC15 (degree = 33), FAM198B‐AS1 (degree = 27) and LINC02544 (degree = 23) were the top three DElncRNAs that covered the most DEmRNAs. GO enrichment analysis revealed that biological processes such as collagen fibril organization (*P* = 4.94E−10), cell adhesion (*P* = 4.83E−4), proteinaceous ECM (*P* = 1.46E−11) and ECM structural constituent (*P* = 1.80E−5) were dysregulated (Fig. S1A–C). KEGG pathway analysis highlighted that focal adhesion (*P* = 1.75E−7), ECM–receptor interaction (*P* = 3.35E−6), glycosaminoglycan biosynthesis‐chondroitin sulfate/dermatan sulfate (*P* = 2.67E−3) and the PI3K–Akt signaling pathway (*P* = 2.79E−3) were dysregulated (Fig. S1D).

### DEcircRNA–DEmRNA coexpression network

**Fig. 4:**
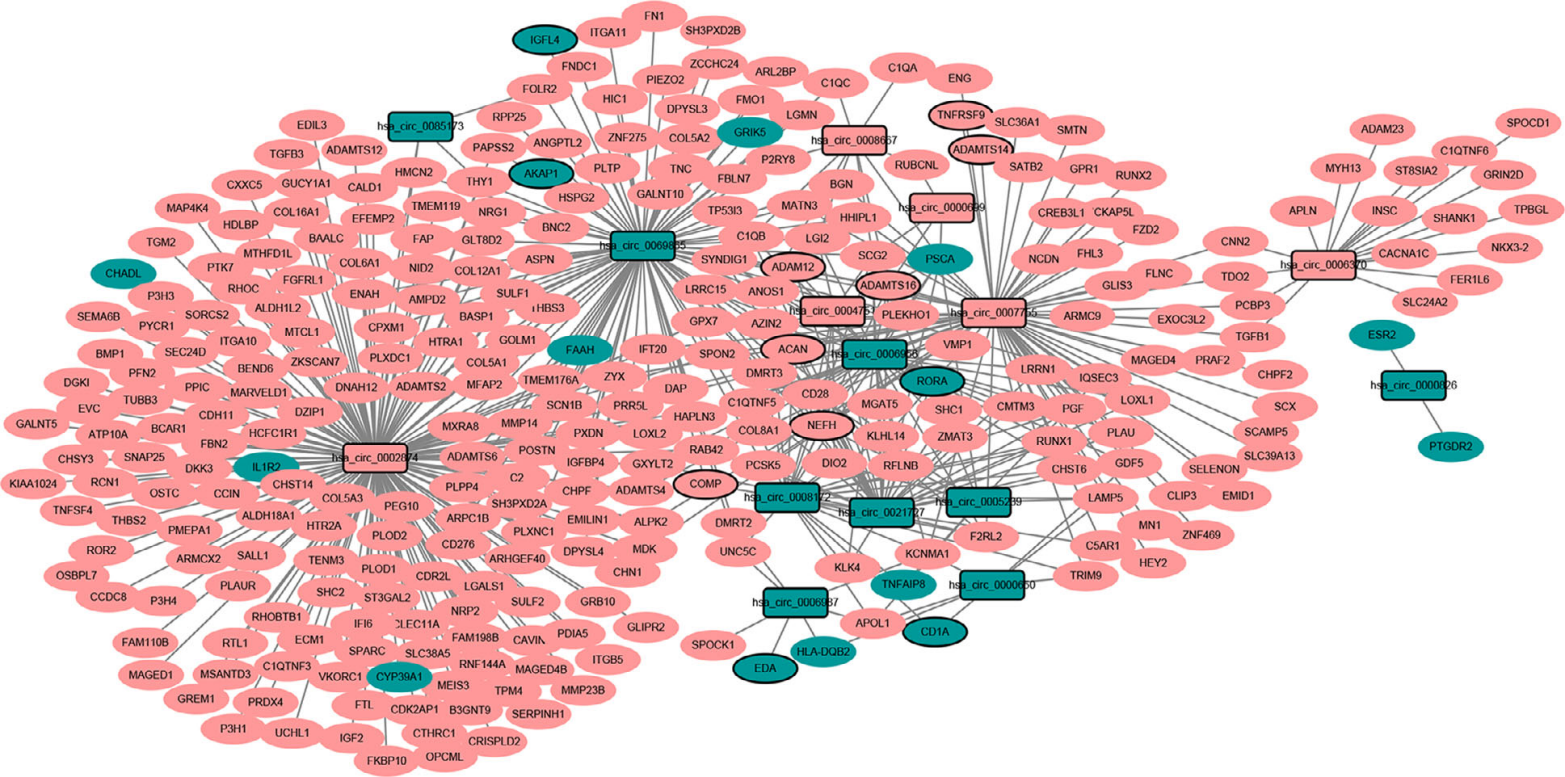
DEcircRNA–DEmRNA coexpression network. The rectangles and ellipses represent DEcircRNAs and DEmRNAs, respectively. Red and blue color represent up‐ and down‐regulation, respectively. Nodes with black border were DEcircRNAs/DEmRNAs derived from top 10 up‐ and down‐regulated DEcircRNAs/DEmRNAs in HS.

A total of 505 DEcircRNA–DEmRNA coexpression pairs, including 15 DEcircRNAs and 299 DEmRNAs, were obtained (Fig. 4). Among these circRNAs, hsa_circ_0002874 (degree = 168), hsa_circ_0069865 (degree = 98) and hsa_circ_0007755 (degree = 73) were the top three DEcircRNAs that covered the most DEmRNAs. GO enrichment analysis revealed that biological processes, such as cell adhesion (*P* = 8.82E−16), ECM organization (*P* = 1.72E−15), proteinaceous ECM (*P* = 3.31E−22), collagen binding (*P* = 1.93E−9) and ECM structural constituent (*P* = 6.59E−9), were dysregulated (Fig. S2A–C). KEGG pathway analysis highlighted that ECM–receptor interaction (*P* = 9.92E−9), focal adhesion (*P* = 2.14E−8), hypertrophic cardiomyopathy (*P* = 1.47E−3) and PI3K–Akt signaling pathway (*P* = 3.26E−3) were dysregulated (Fig. S2D).

### ceRNA (DEcircRNA/DElncRNA–miRNA–DEmRNA) regulatory network

**Fig. 5:**
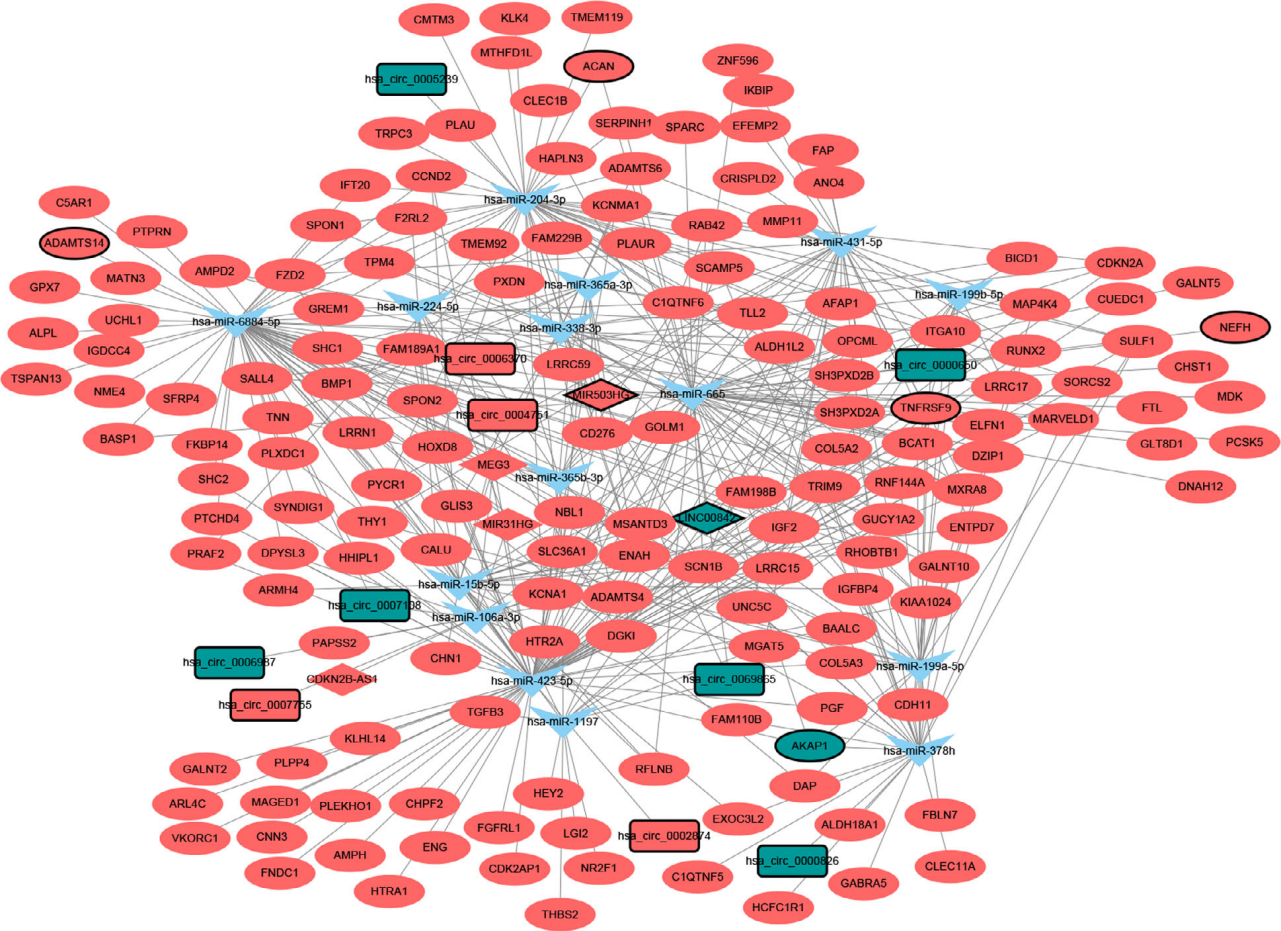
ceRNA (DEcircRNA/DElncRNA–miRNA–DEmRNA) regulatory network. The rectangles, rhombuses, inverted triangles, and ellipses indicate DEcircRNAs, DElncRNAs, miRNAs and DEmRNAs, respectively. Red and blue colors represent up‐regulation and down‐regulation, respectively. Nodes with black border were DEcircRNAs/DElncRNAs/DEmRNAs derived from top 10 up‐ and down‐regulated DEcircRNAs/DElncRNAs/DEmRNAs in HS.

The ceRNA network contained 18 circRNA–miRNA pairs, 18 lncRNA–miRNA pairs and 409 miRNA–mRNA pairs, including 10 circRNAs, 5 lncRNAs, 15 miRNAs and 160 mRNAs (Fig. 5). For DEmRNAs involved in the ceRNA network, collagen fibril organization (*P* = 6.18E−6), proteinaceous ECM (*P* = 3.41E−06), ECM structural constituent (*P* = 2.25E−4), focal adhesion (*P* = 3.10E−4), ECM–receptor interaction (*P* = 6.05E−3) and PI3K–Akt signaling pathway (*P* = 6.34E−2) were several significantly enriched pathways (Fig. S3D).

## Discussion

HS, a fibroproliferative disorder, is characterized by excessive deposition of ECM and invasive growth of fibroblasts [12]. In this study, a total of 487 DEmRNAs, 92 DElncRNAs and 17 DEcircRNAs were identified in HS. In addition, several pathways were identified to be closely associated with HS, including collagen fibril organization, ECM–receptor interaction and PI3K–Akt signaling pathway. According to the results of functional annotation analysis, *ADAMTS14*, *ACAN* and *COL11A1* were enriched in collagen fibril organization; *COMP* was enriched in ECM–receptor interaction; and *COMP* and *COL11A1* were enriched in the PI3K–Akt signaling pathway.

Collagen is the main component of interstitial ECM, which is involved in the regulation of various biological processes, such as cell morphology, proliferation, migration, differentiation, apoptosis and carcinogenesis [13]. As a member of minor fibrillar collagens, COL11A1 can be produced by cartilage and a variety of noncartilaginous tissues, including skin [14]. Recent studies have found that *COL11A1* is associated with various cancers, such as gastric cancer, ovarian cancer and non‐small cell lung cancer [15, 16, 17]. It has been concluded that *COL11A1* expression is a biomarker of human carcinoma‐associated stromal cells and carcinoma progression [18]. *COL11A1* has been found to be overexpressed in human keloid fibroblasts related to normal skin fibroblasts [19]. Elevated *COL11A1* was also observed in scleroderma skin, another condition with extensive fibroblast activation [20]. *P4HA3* encodes a component of prolyl 4‐hydroxylase, a key enzyme in collagen synthesis. In general, the expression of *P4HA3* is very low in normal fetal and adult tissues [21]. In poorly differentiated gastric adenocarcinoma cancer cell line MKN‐45 and AGS cells, up‐regulated *P4HA3* could enhance cell motility and invasiveness [22]. Highly expressed *P4HA3* was associated with poor prognosis in gastric cancer [23]. There were no previous reports of the function of *P4HA3* in HS. Here, significantly up‐regulated *COL11A1* and *P4HA3* were observed in HS, adding evidence that *COL11A1* and *P4HA3* may play an important role in HS.

*CD1A* encodes a member of the CD1 family of transmembrane glycoproteins, which are structurally related to the major histocompatibility complex proteins and form heterodimers with β_2_‐microglobulin. The amount of CD1As of the positive dendritic cell was significantly higher in HS than the controlled normal skin [24]. However, *CD1A* was identified to be the most significantly down‐regulated DEmRNA in this study, indicating that its exact role in HS needs further study to determine. A recent study indicated that LINC01189 was significantly altered in peripheral blood mononuclear cells of patients with rheumatoid arthritis, suggesting that LINC01189 may be a potential biomarker for rheumatoid arthritis [25]. Besides, Yao *et al*. [26] demonstrated that LINC01189 was down‐regulated in hepatitis C virus‐infected hepatocellular carcinoma (HCC) tumors and cell lines and may confer a suppression effect on the development of HCC. Other than that, the expression pattern or regulatory effects of LINC01189 in other human diseases have never been elucidated. In other words, LINC01189, the most significantly down‐regulated DElncRNA, was first reported to be associated with HS in this study.

Aggrecan, encoded by *ACAN*, is a major proteoglycan component in the ECM of the growth plate and articular cartilage [27]. Mutations in *ACAN* were reported to be associated with growth defects ranging from mild idiopathic short stature to severe skeletal dysplasias [28]. *TNFRSF9*, also termed *4‐1BB* and *CD137*, is a member of the tumor necrosis factor receptor superfamily, which contributes to the clonal expansion, survival, and development of T cells. It has been suggested that *TNFRFS9* expression was a biomarker for tumor‐infiltrating lymphocytes in ovarian cancer and melanoma [29]. In addition, *TNFRSF9* methylation has been reported to serve as a biomarker in the context of immunotherapies in melanoma [30]. Recently, lncRNA MIR503HG has been suggested to be dysregulated and involved in a variety of human cancers. Qiu *et al*. [31] suggested that MIR503HG exhibited significant antiproliferation and antimigration/invasion effects on bladder cancer cells. Chuo *et al*. [32] demonstrated that MIR503HG overexpression inhibits colorectal cancer cell migration and invasion mediated by transforming growth factor‐β2. Lin *et al*. [33] revealed that MIR503HG suppressed non‐small cell lung cancer progression via negatively regulating Wnt1 expression. *ACAN*, *TNFRSF9* and MIR503HG were identified to be dysregulated in this study, although no previous study linked *ACAN*, *TNFRSF9* and MIR503HG with HS. In addition, *ACAN* and *TNFRSF9* were targets of MIR503HG (MIR503HG/hsa‐miR‐204‐3p/*ACAN* and MIR503HG/hsa‐miR‐431‐5p/TNFRSF9) in the ceRNA network, which indicated that MIR503HG may act as a ceRNA to regulate the expression of ACAN and TNFRSF9 in HS.

*ADAMTS14*, located on chromosome 10q22.1, is a member of the ADAMTS metalloproteinase family, comprised of 19 members, which are known as proteolytic enzymes to catalyze a great variety of substrates in the ECM [34]. The activation of ADAMTS proteinases can exhibit both inhibitory and promotive effects on angiogenesis because the mechanism involved in their regulation of cancer development varies among different members [35]. Sheu *et al*. [36] implicated the *ADAMTS14* gene polymorphism as a predictive factor of HCC. Low cytoplasmic expression of *ADAMTS14* has been associated with poor overall survival of patients with oral squamous cell carcinoma, which may be used as a novel biomarker for oral squamous cell carcinoma diagnosis [37]. MEG3, located on chromosome 14q32.3, has been associated with various tumors and regarded as a putative cancer biomarker and treatment target [38]. In the ceRNA network, *ADAMTS14* was a target of MEG3 (MEG3/hsa‐miR‐6884‐5p/*ADAMTS14*). In the DElncRNA–DEmRNA coexpression network, *ADAMTS14* was coexpressed with AC000035.1 (one of the top 10 significantly up‐regulated DElncRNAs). Hence we speculated that MEG3 and AC000035.1 may participate in HS via regulating *ADAMTS14*.

*COMP* is a fibrillar collagen assembly regulator, which is involved in the assembly and stabilization of the ECM via its interactions with type I and type II collagen and modulates the cellular phenotype during tissue genesis and remodeling [39, 40]. Zachou *et al*. [41] suggested *COMP* as a biomarker of liver fibrosis in patients with chronic viral hepatitis. Li *et al*. [39] reported that hepatic stellate cell‐derived *COMP* drives HCC progression by activating mitogen‐activated protein kinase kinase 7 (MEK)/mitogen‐activated protein kinase (ERK) and PI3K/AKT signaling pathways. Vuga *et al*. [42] demonstrated that *COMP* may serve as a biomarker for idiopathic pulmonary fibrosis. Agarwal *et al*. [43] indicated that COMP is also a constitutive component present in human skin that is deposited by fibroblasts into the ECM of human skin. Agarwal *et al*. [44] recently demonstrated that COMP deposition is enhanced in the dermis in various fibrotic conditions. *ADAM12* encodes a member of the a disintegrin and metalloprotease (ADAM) protein family and is restrictively expressed in normal tissues [45]. It was reported that up‐regulated *ADAM12* in the central part of keloids may be involved in processes leading to clinical regression [19]. In the DEcircRNA–DEmRNA coexpression network, hsa_circ_0069865, one of the top three down‐regulated DEcircRNAs that covered the most DEmRNAs, was coexpressed with *COMP* and *ADAM12*, which may suggest hsa_circ_0069865 was involved in HS mediated by *COMP* and *ADAM12*.

## Conclusion

We highlighted the roles of several DEmRNAs (*COL11A1*, *P4HA3*, *CD1A*, *ACAN*, *TNFRSF9*, *ADAMTS14*, *ADAM12* and *COMP*) and six interaction pairs (MIR503HG/hsa‐miR‐204‐3p/*ACAN*, MIR503HG/hsa‐miR‐431‐5p/*TNFRSF9*, MEG3/hsa‐miR‐6884‐5p/*ADAMTS14*, AC000035.1–*ADAMTS14* and hsa_circ_0069865‐*COMP*/*ADAM12*) in HS. This work may aid identification of prospective clinical markers and is expected to contribute to understanding the pathophysiology of HS, and further studies with larger sample size are warranted to confirm these results.

## Conflict of interest

The authors declare no conflict of interest.

## Author contributions

XL and YH contributed to the study conception and design. Material preparation, data collection and analysis were performed by XL, ZH and JZ. The first draft of the manuscript was written by XL, and all authors commented on previous versions of the manuscript. All authors read and approved the final manuscript.

## Supporting information


**Fig. S1**. Significantly enriched GO terms and KEGG pathways of DEmRNAs in DElncRNA–DEmRNA coexpression network. (A) BP, biological process; (B) CC, cellular component; (C) MF, molecular function; (D) KEGG pathways. The *x* axis shows *P* value of GO terms or KEGG pathways and the *y* axis shows GO terms or KEGG pathways. The color scale represented −log *P* value.

**Fig. S2**. Significantly enriched GO terms and KEGG pathways of DEmRNAs in DEcircRNA–DEmRNA coexpression network. (A) BP, biological process; (B) CC, cellular component; (C) MF, molecular function; (D) KEGG pathways. The *x* axis shows *P* value of GO terms or KEGG pathways, and the *y* axis shows GO terms or KEGG pathways. The color scale represented −log *P* value.

**Fig. S3**. Significantly enriched GO terms and KEGG pathways of DEmRNAs in the ceRNA network. (A) BP, biological process; (B) CC, cellular component; (C) MF, molecular function; (D) KEGG pathways. The *x* axis shows *P* value of GO terms or KEGG pathways, and the *y* axis shows GO terms or KEGG pathways. The color scale represented −log *P* value.

## Data Availability

The datasets used and/or analyzed during this study are available from the corresponding author on reasonable request.
