# Supplementary figures and images for: Identification of crucial noncoding RNAs and mRNAs in hypertrophic scars via RNA sequencing
Source: FEBS Open Bio. 2021 May 12;11(6):1673–84. doi: 10.1002/2211-5463.13167 (PMC8167876; doi:10.1002/2211-5463.13167)

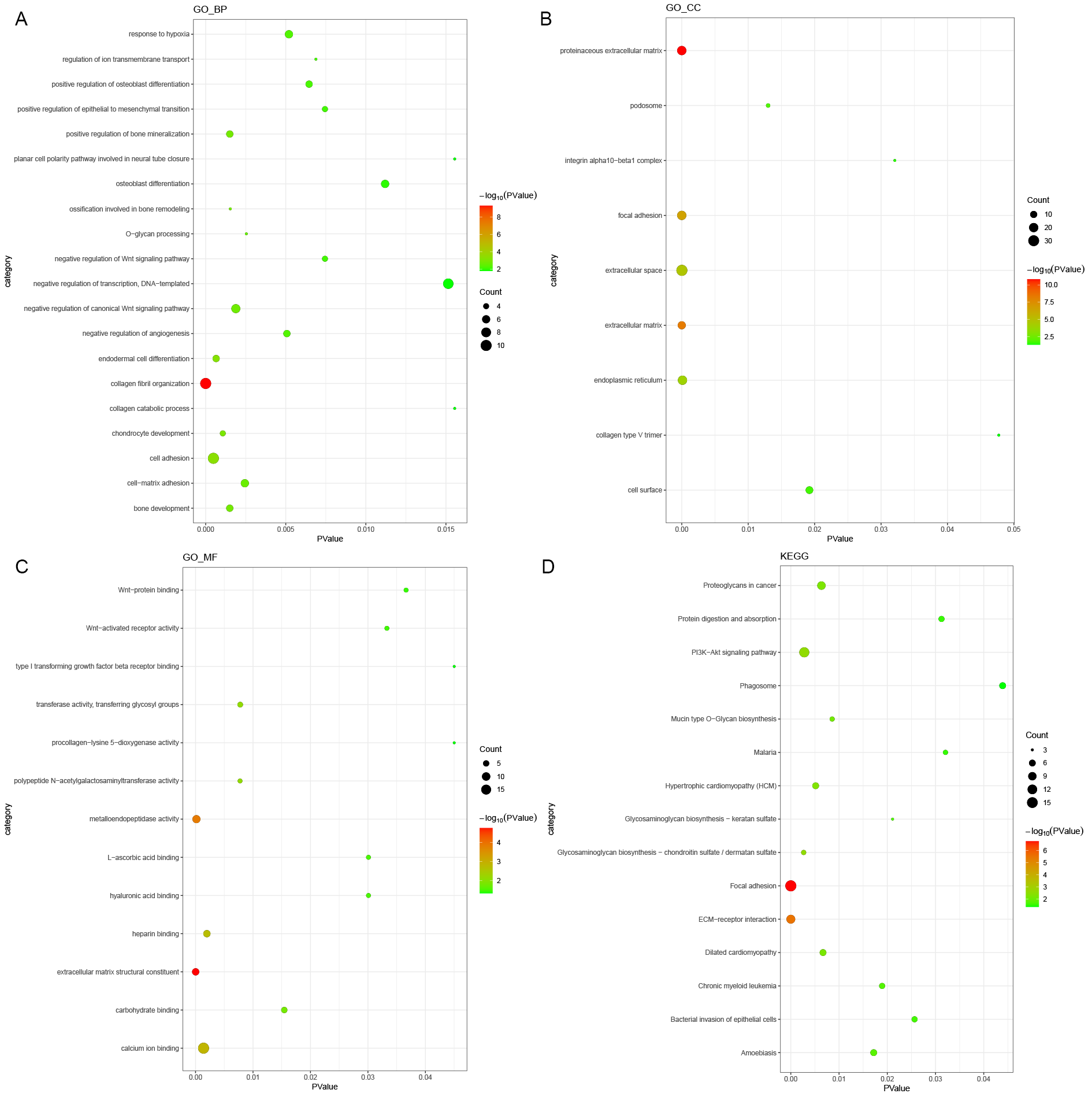

Supplement: Supplementary file 1 — Fig. S1. Significantly enriched GO terms and KEGG pathways of DEmRNAs in DElncRNA–DEmRNA coexpression network. (A) BP, biological process; (B) CC, cellular component; (C) MF, molecular function; (D) KEGG pathways. The x axis shows P value of GO terms or KEGG pathways and the y axis shows GO terms or KEGG pathways. The color scale represented −log P value. [file FEB4-11-1673-s001.tif]

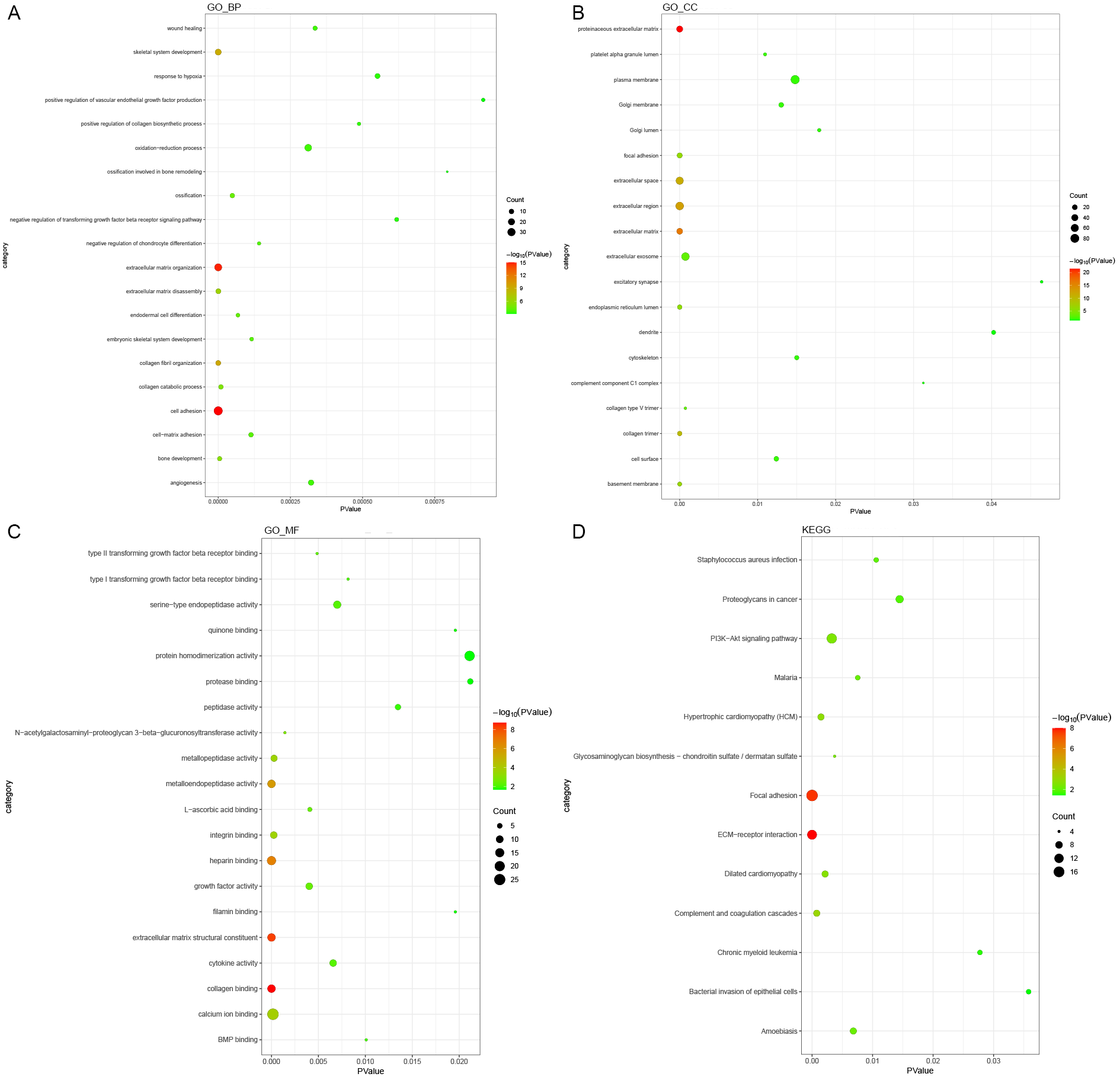

Supplement: Supplementary file 2 — Fig. S2. Significantly enriched GO terms and KEGG pathways of DEmRNAs in DEcircRNA–DEmRNA coexpression network. (A) BP, biological process; (B) CC, cellular component; (C) MF, molecular function; (D) KEGG pathways. The x axis shows P value of GO terms or KEGG pathways, and the y axis shows GO terms or KEGG pathways. The color scale represented −log P value. [file FEB4-11-1673-s003.tif]

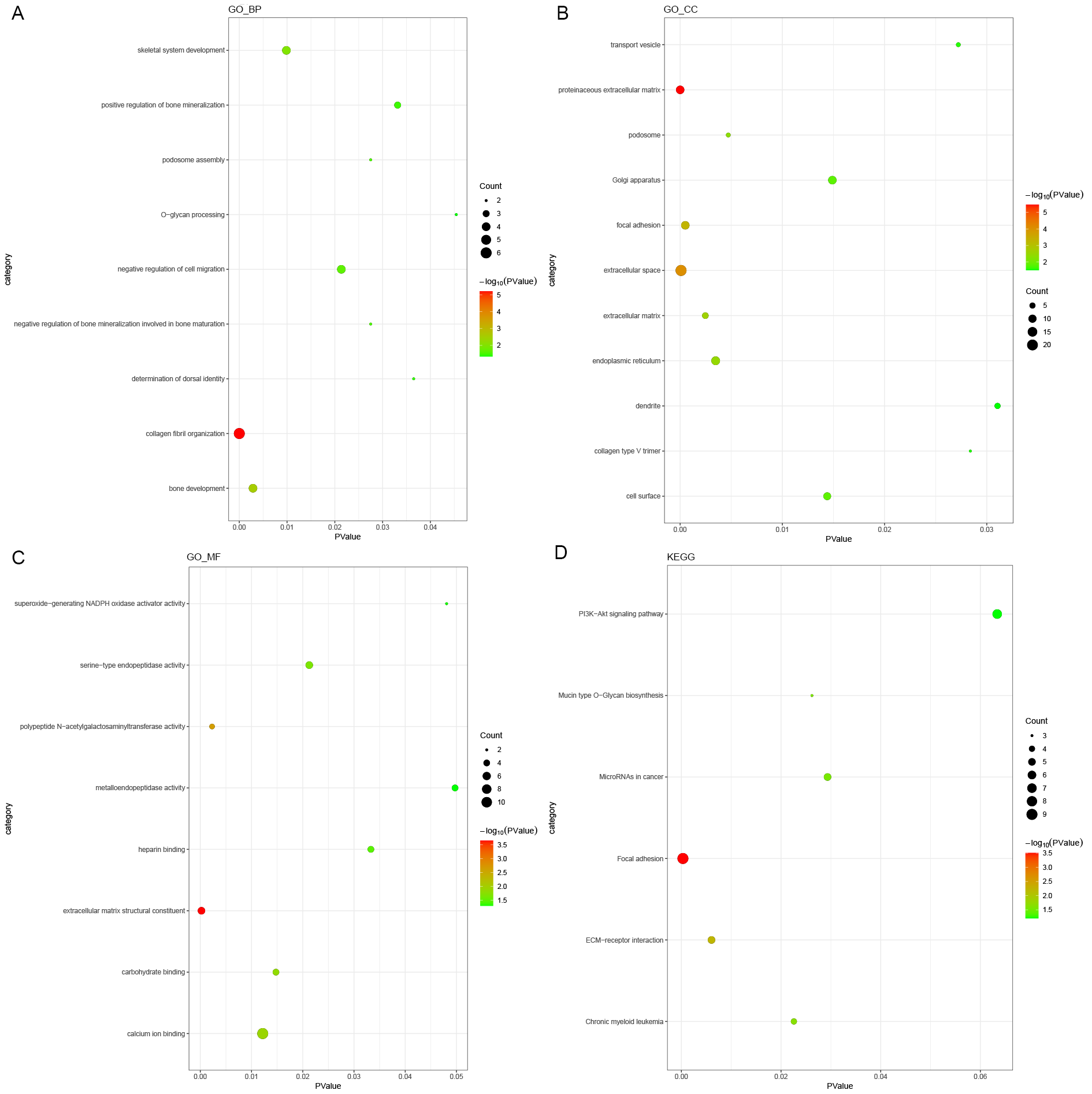

Supplement: Supplementary file 3 — Fig. S3. Significantly enriched GO terms and KEGG pathways of DEmRNAs in the ceRNA network. (A) BP, biological process; (B) CC, cellular component; (C) MF, molecular function; (D) KEGG pathways. The x axis shows P value of GO terms or KEGG pathways, and the y axis shows GO terms or KEGG pathways. The color scale represented −log P value. [file FEB4-11-1673-s002.tif]
